# Optimizing ultrafast dynamic contrast-enhanced MRI scan duration in the differentiation of benign and malignant breast lesions

**DOI:** 10.1186/s13244-024-01697-6

**Published:** 2024-05-07

**Authors:** Ying Cao, Yao Huang, Xianglong Chen, Wei Wang, Huifang Chen, Ting Yin, Dominik Nickel, Changchun Li, Junhua Shao, Shi Zhang, Xiaoxia Wang, Jiuquan Zhang

**Affiliations:** 1 School of Medicine, Chongqing University, Chongqing, China; 2 Department of Radiology, Chongqing University Cancer Hospital, Chongqing Key Laboratory for Intelligent Oncology in Breast Cancer (iCQBC), Chongqing, China; 3 School of Medical Imaging, North Sichuan Medical University, Nanchong, China; 4 MR Collaborations, Siemens Healthineers Ltd., Chengdu, China; 5 MR Application Predevelopment, Siemens Healthcare GmbH, Erlangen, Germany

**Keywords:** Breast neoplasms, Magnetic resonance imaging, Kinetics, Differential diagnosis

## Abstract

**Objective:**

To determine the optimal scan duration for ultrafast DCE-MRI in effectively differentiating benign from malignant breast lesions.

**Methods:**

The study prospectively recruited participants who underwent breast ultrafast DCE-MRI from September 2021 to March 2023. A 30-phase breast ultrafast DCE-MRI on a 3.0-T MRI system was conducted with a 4.5-s temporal resolution. Scan durations ranged from 40.5 s to 135.0 s, during which the analysis is performed at three-phase intervals, forming eight dynamic sets (scan duration [SD]_40.5s_: 40.5 s, SD_54s_: 54.0 s, SD_67.5s_: 67.5 s, SD_81s_: 81.0 s, SD_94.5s_: 94.5 s, SD_108s_: 108.0 s, SD_121.5s_: 121.5 s, and SD_135s_: 135.0 s). Two ultrafast DCE-MRI parameters, maximum slope (MS) and initial area under the curve in 60 s (iAUC), were calculated for each dynamic set and compared between benign and malignant lesions. Areas under the receiver operating characteristic curve (AUCs) were used to assess their diagnostic performance.

**Results:**

A total of 140 women (mean age, 47 ± 11 years) with 151 lesions were included. MS and iAUC from eight dynamic sets exhibited significant differences between benign and malignant lesions (all *p* < 0.05), except iAUC at SD_40.5s_. The AUC of MS (AUC = 0.804) and iAUC (AUC = 0.659) at SD_67.5s_ were significantly higher than their values at SD_40.5s_ (AUC = 0.606 and 0.516; corrected *p* < 0.05). No significant differences in AUCs for MS and iAUC were observed from SD_67.5s_ to SD_135s_ (all corrected *p* > 0.05).

**Conclusions:**

Ultrafast DCE-MRI with a 67.5-s scan duration appears optimal for effectively differentiating malignant from benign breast lesions.

**Critical relevance statement:**

By evaluating scan durations (40.5–135 s) and analyzing two ultrafast DCE-MRI parameters, we found a scan duration of 67.5 s optimal for discriminating between these lesions and offering a balance between acquisition time and diagnostic efficacy.

**Key Points:**

- Ultrafast DCE-MRI can effectively differentiate malignant from benign breast lesions.
- A minimum of 67.5-sec ultrafast DCE-MRI scan duration is required to differentiate benign and malignant lesions.
- Extending the scan duration beyond 67.5 s did not significantly improve diagnostic accuracy.

**Graphical Abstract:**

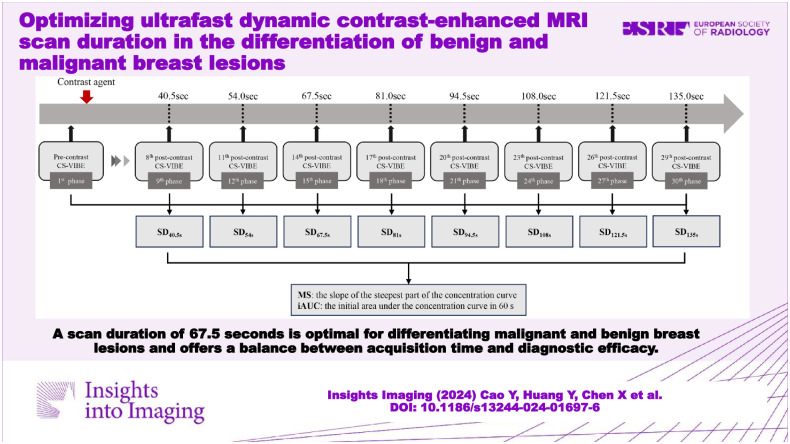

## Introduction

Breast cancer is currently the most prevalent cancer in women worldwide, and has an increasing incidence [1]. Accurate differential diagnosis of breast lesions is of paramount clinical importance as it directly influences patient management, treatment decisions, and prognosis. Among the various imaging modalities employed in differentiating benign from malignant breast lesions, dynamic contrast-enhanced magnetic resonance imaging (DCE-MRI) has become the most sensitive modality over the past two decades [2]. Remarkably, DCE-MRI excels, especially in the realm of malignancy exclusion, with a consistently high negative predictive value (NPV) typically exceeding 90% [3–5]. The conventional kinetic analysis in DCE-MRI involves constructing time-intensity curves based on pre-contrast, initial, and delayed phase images to improve the specificity in assessing breast lesions, ending up with a considerable scan time commitment of 6–10 min [6]. As a result, the prolonged scan duration of DCE-MRI scans has limited its routine clinical use and broader applications [7, 8].

Ultrafast DCE-MRI has been introduced as a relatively novel imaging technique that documents the early influx of contrast agents with exceptional temporal resolution while preserving necessary diagnostic spatial resolution [9, 10]. This technique allows for acquiring images at multiple time points, particularly in the early post-contrast phase (≤ 2 min), and generates a plurality of kinetic parameters reflective of wash-in. Previous studies have reported promising results of ultrafast DCE-MRI parameters in discriminating between benign and malignant breast lesions [11–14]. For instance, Onishi et al demonstrated that the initial area under gadolinium contrast agent concentration (IAUGC) was significantly greater in breast cancer compared to benign lesions [12]. Additionally, Mann et al suggested that the maximum slope (MS) of malignant breast lesions is higher than that of benign lesions, providing a basis for differentiating malignant from benign breast lesions [13].

Despite these advancements, the standardization of ultrafast DCE-MRI scan duration remains unresolved. Currently, the acquisition times vary across institutions and studies, ranging from 60 s to 135 s (Table 1) [11, 12, 14–25]. A comparative analysis of these varying scan durations within the same cohort is needed to identify the optimal time frame for effectively discriminating between benign and malignant breast lesions.

**Table 1:** Summary of ultrafast DCE-MRI scan duration in different studies

Reference (year)	Author	Sequence	Temporal resolution	Scan duration	Parameter/model	AUC
[14] (2023)	MT Ramli Hamid et al	Ultrafast TWIST	3.7 s	75 s	MS, TTE, AVI	MS = 0.836, TTE = 0.647, AVI = 0.684
[18] (2023)	Ying Cao et al	CS-VIBE	4.5 s	135 s	MS, TTP, TTE, iAUC	TTP = 0.826, MS = 0.751, TTE = 0.721, iAUC = 0.577
[19] (2023)	Yidong Lyu et al	DISCO	4.8 s	135 s	ANN models	0.915–0.956
[17] (2021)	Margaux Pelissier et al	TWIST-VIBE	7.1 s	78 s	MS	0.94
[12] (2020)	Natsuko Onishi et al	DISCO	2.7–7.1 s	60 s	MS, CER, BAT, IAUGC	MS + BAT + age model = 0.846 MS + BAT model = 0.704
[15] (2020)	Sandra C Peter et al	TVD	4.9 s	98 s	TVD model	0.938
[20] (2020)	Maya Honda et al	CS-VIBE	3.7 s	75 s	MS, TTE, AVI	MS = 0.76, TTE = 0.78, AVI = 0.76
[21] (2020)	Akane Ohashi et al	CS-VIBE	3.7 s	75 s	MS	0.74
[24] (2020)	Soo Jeong Lee et al	DISCO	6.5 s	80 s	IER, slope_max_, ME, slope, PMS	IER = 0.800, slope_max_ = 0.748, ME = 0.748, PMS = 0.665
[11] (2019)	Mariko Goto et al	TWIST-VIBE	5.3 s	107 s	TTE, MS	NME group: MS + TTE + BI-RADS = 0.86 Masses group: MS + TTE + BI-RADS = 0.92
[22] (2019)	Chengyue Wu et al	Spoiled GRE	Group A: 3.4–4.1 s Group B: 1.7–3.5 s	Group A: 90 s Group B: 42 s	BAT, *K*_trans_, *V*_p_	Vessel count + BAT = 0.91
[23] (2019)	Akane Ohashi et al	KWIC	3.75 s	60 s	MS	0.81
[25] (2018)	Natsuko Onishi et al	VIBE	3.65 s	73 s	AVI	/
[16] (2017)	Roel D Mus et al	TWIST	4.32 s	102 s	TTE	Reader1 = 0.86, reader2 = 0.80

*TWIST* time-resolved angiography with stochastic trajectories, *CS* compressed sensing, *VIBE* volume-interpolated breath-hold examination, *DISCO* differential sub-sampling with cartesian ordering, *TWIST* time-resolved angiography with interleaved stochastic trajectories, *TVD* TWIST-VIBE Dixon, *GRE* gradient recalled echo protocol, *KWIC* κ-space-weighted image contrast sequence, *MS* maximum slope, *TTE* time-to-enhancement, *AVI* time interval between arterial and venous visualization, *TTP* time-to-peak, *iAUC* initial area under the curve in 60 s, *ANN* artificial neural network, *CER* contrast enhancement ratio, *BAT* bolus arrival time, *IAUGC* initial area under gadolinium contrast agent concentration, *IEP* initial enhancement phase by reviewer, *ME* maximum enhancement, *PMS* the phase with slope_max_, *K*_*trans*_ volume transfer coefficient, *V*_*p*_ plasma volume fraction

Therefore, our study seeks to explore how different scan durations in ultrafast DCE-MRI affect the performance of its derived parameters in distinguishing between benign and malignant breast lesions. The goal is to establish the optimal scan duration that minimizes acquisition time while retaining robust discriminatory ability.

## Materials and methods

### Participants

**Fig. 1:**
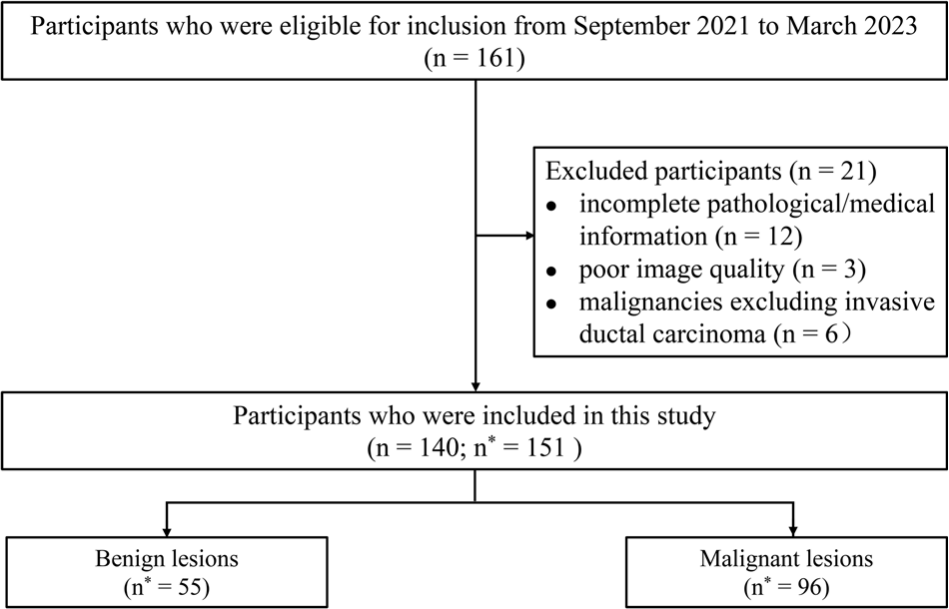
Flow diagram of participant selection. *n* Represents the number of participants, and n^*^ represents the number of lesions

This prospective study received approval from our institutional review committee, and all participants provided written informed consent for their involvement. The study was conducted from September 2021 to March 2023, during which eligible participants were enrolled. The indications for performing MRI were based on clinical criteria [26–28] such as (i) diagnosis and preoperative assessment: suspicious mammography and/or ultrasound findings, palpable breast masses, or screening for multi-focal lesions; (ii) screening for populations with high-risk factors of breast cancer, including family history, dense breast tissue, breast abnormalities, or symptoms suggestive of breast biopsy. The inclusion criteria for this study were as follows: (i) suspicious findings on mammography and/or ultrasound [Breast Imaging Reporting and Data System [28] (BI-RADS) categories ≥ 4]; (ii) no history of breast surgery before MRI examination; (iii) no prior biopsies or previous treatment for breast cancer before MRI examination; and (iv) pathological confirmation of the breast lesions as benign or malignant. The exclusion criteria included: (i) incomplete pathological/medical information (*n* = 12); (ii) poor image quality (*n* = 3); and (iii) pathological confirmed malignancies other than invasive ductal carcinoma (*n* = 6). Finally, 140 participants with 151 lesions were included, with 11 subjects presenting bilateral lesions. The flowchart of patient selection is shown in Fig. 1.

### Ultrafast MRI acquisition protocol

The MRI scans were conducted utilizing a 3 Tesla MRI system (MAGNETOM Prisma, Siemens Healthineers, Erlangen, Germany) with a dedicated 16-channel bilateral breast coil. All participants were imaged in a head-first prone position. Breast compression was not applied. The MRI protocol included routine axial T1-weighted, T2-weighted, diffusion-weighted, and ultrafast DCE-MRI with a T1 map. The ultrafast DCE-MRI was performed with a research application using a compressed sensing (CS) accelerated T1-weighted gradient echo sequence with Dixon water-fat separation based on the volumetric interpolated breath-hold examination sequence (VIBE) with the following protocol parameters: repetition time (TR) = 4.46 msec, echo times (TE) = 1.55/2.73 msec, flip angle = 11°, field of view (FOV) = 360 × 292 mm^2^, voxel size = 0.9 × 0.9 × 2.5 mm^3^, bandwidth = 810 Hz/Px. One pre-contrast and 29 post-contrast phases of ultrafast DCE-MRI were acquired at a temporal resolution of 4.5 s/phase. To calculate the tissue concentration curve, a two-flip angle T1 map using the VIBE sequence was obtained prior to the DCE scans: TR/TE = 5.03/1.79 msec, flip angle = 2°/10°, FOV = 360 × 360 mm^2^, voxel size = 1.4 × 1.4 × 2 mm^3^, and bandwidth = 260 Hz/Px. The contrast agent used was Gadobutrol meglumine (Jia Di Xian^®^, Heng Rui), infused intravenously at a dose of 0.2 mL/kg (0.1 mmol/kg) and at a speed of 2.0 mL/s, followed by a 20-mL saline flush at the same rate.

### Image analysis

**Fig. 2:**
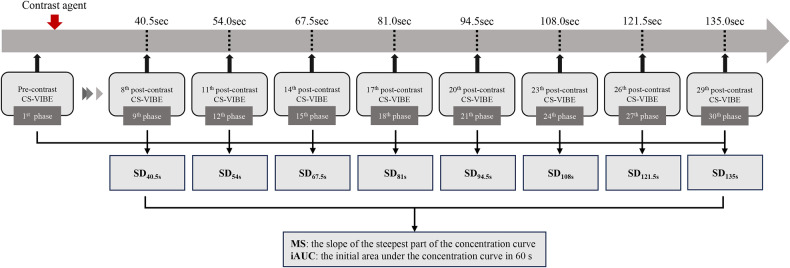
Flow chart of ultrafast DCE-MRI data processing. The image set that showed the pre-contrast CS-VIBE was marked as the first phase. The pre-contrast sequences and different phases of post-contrast sequences (1–9, 1–12, 1–15, 1–21, 1–24, 1–27, and 1–30) were used to form eight sets of dynamic series with scan duration ranging from 40.5 s t0 135.0 s. CS-VIBE T1-weighted compressed-sensing volume interpolated breath-hold examination

The T1 maps and DCE data were uploaded to a post-processing workstation for semi-quantitative analysis using Tissue 4D software (Siemens Healthineers. Erlangen, Germany). Acknowledging the variable scan durations observed in prior studies, ranging from 60 s to 135 s [11, 12, 14–25], we tailored the scan duration ranging from 40.5 s (4.5 s × 9 phases) to 135 s (4.5 s × 30 phases). The imaging protocol was designed to consist of one pre-contrast CS-VIBE phase combined with different post-contrast phases (1–9, 1–12, 1–15, 1–18, 1–21, 1–27, and 1–30), forming a total of eight dynamic datasets. Each dataset corresponded to specific scan durations of 40.5 s (SD_40.5s_), 54 s (SD_54s_), 67.5 s (SD6_7.5s_), 81 s (SD_81s_), 94.5 s (SD_94.5s_), 108 s (SD_108s_), 121.5 s (SD_121.5s_), and 135 s (SD_135s_) as shown in Fig. 2.

To obtain the ultrafast DCE-MRI parameters for each lesion, two radiologists (W.W. and X.C. with seven and six years of experience in breast MRI, respectively) blinded to pathological reports independently outlined the tumor volumes of interest (VOIs). In case of discrepancies between the delineations made by the two radiologists, a consensus approach was adopted to reach a final agreement. The VOIs were drawn on the central slice of the tumor’s largest section and automatically extended to surrounding slices to form a spherical VOI [29]. The radiologists then refined these volumetric segmentations as needed, avoiding areas of liquefaction, necrosis, or cystic changes. To ensure an accurate delineation of lesion contours, for each dynamic set, the VOIs were drawn at the frame with the most obvious tumor enhancement. In cases where lesions did not exhibit early-phase enhancement, VOIs were positioned taking the 30th frame of the DCE scan as reference.

We focused on two key parameters from ultrafast DCE-MRI: MS and initial area under the curve in 60 s (iAUC). MS was defined as the slope of the concentration curve’s steepest segment. iAUC represented the initial area under the concentration curve after 60 s of contrast injection, except for SD_40.5s_ and SD_54s_ (hereafter referred to as iAUC_40.5s_ and iAUC_54s_, respectively), in which the iAUC was calculated only till the last scan timepoint. Both parameters were independently recorded for each lesion by the radiologists. Considering the senior radiologist’s extensive experience and expertize in interpreting DCE-MRI images for breast lesions, the data from the senior radiologist were used for statistical analysis. Additionally, to assess intra-observer agreement, the senior radiologist re-evaluated 30 randomly selected participants after three months.

### Clinicopathologic information

Data collected from the medical record were participant age (years), menopausal status, histological type, estrogen/progesterone receptor status, human epidermal growth factor receptor 2 (HER2) status, Ki67 status, lymph node metastasis, and lesion size (mm) [30, 31]. Lesions were categorized as benign or malignant based on pathology, with invasive ductal carcinoma classified as “malignant” and breast mastitis and other benignancies classified as “benign”.

### Statistical analysis

Statistical analyses were conducted using the SPSS (version 26, IBM) and MedCalc software (version 20.022, MedCalc). The normality of ultrafast DCE-MRI parameters was tested using the Kolmogorov–Smirnov test. Differences in ultrafast DCE-MRI parameters between benign and malignant groups were compared using the Student’s *t* test if normally distributed and the Mann–Whitney *U* test if non-normally distributed. Paired *t* tests with Bonferroni correction were employed to discern significant differences in these parameters for benign and malignant breast lesions among eight scan durations, assuming a normal distribution. Nonparametric Cochran’s *Q* tests were utilized otherwise. Diagnostic performance was evaluated via receiver-operating characteristic (ROC) curve analysis, with the area under the ROC curve (AUC) calculated and compared using the DeLong test. Sensitivity, specificity, positive predictive value (PPV), NPV, and accuracy were also calculated. Additionally, the cutoff values were determined using the Youden index. The inter- and intra-observer agreements for the ultrafast DCE-MRI parameters were evaluated using intraclass correlation coefficients (ICCs). Statistical significance was considered at *p* < 0.05.

## Results

### Participant characteristics

In total, 151 pathologically proven breast lesions in 140 women (mean age, 47 ± 11 years; range, 19–77 years) were analyzed. Among these participants, 129 had a single lesion, and 11 had bilateral lesions. Table 2 summarizes the detailed clinicopathological information of the participants. The 151 lesions were pathologically diagnosed as 55 benignancy (36%) and 96 malignancy (64%). Of the 55 benign lesions, 39 (70%) were fibroadenomas, six (11%) were adenoses, two (4%) were hyperplasia, one (2%) were phyllodes tumors, two (4%) were intraductal papilloma, and five (9%) were mastitis. All the 96 malignant lesions (100%) were invasive ductal carcinomas (IDC). The mean sizes of benign and malignant tumors were 22.3 ± 11.7 mm and 31.3 ± 15.5 mm, respectively, with three lesions (two benign and one malignant) being smaller than 10 mm.

**Table 2:** Participant and tumor characteristics

Characteristics	Datum
Age, mean ± SD, years (range)^a^	
Benignancy	40 ± 10 years (range, 19–63 years)
Malignancy	50 ± 9 years (range, 26–77 years)
Menopausal status (n^*^ = 140)	
Premenopausal women	74 (53)
Postmenopausal women	66 (47)
Benign lesions (*n* = 55)	
Fibroadenoma	39 (70)
Adenosis	6 (11)
Hyperplasia	2 (4)
Phyllodes tumor	1 (2)
Intraductal papilloma	2 (4)
Mastitis	5 (9)
Malignant lesions (*n* = 96)	
Invasive ductal carcinoma	96 (100)
Estrogen receptor status (*n* = 96)	
Positive	54 (56)
Negative	42 (44)
Progesterone receptor status (*n* = 96)	
Positive	35 (36)
Negative	61 (64)
HER2 status (*n* = 96)	
Positive	50 (52)
Negative	46 (48)
Ki67 status (*n* = 96)	
Positive	73 (76)
Negative	23 (24)
Lymph node metastasis (*n* = 96)	
Positive	71 (74)
Negative	25 (26)
Molecular subtype (*n* = 96)	
Luminal A	8 (8)
Luminal B	47 (49)
HER2-enriched	10 (11)
Triple-negative	31 (32)
Lesion size (mm)^a^	
Benignancy	22.3 ± 11.7 mm (range, 9–57 mm)
Malignancy	31.3 ± 15.5 mm (range, 7–106 mm)
MRI BI-RADS classification (*n* = 151)	
3	11
4	98
5	42

Note: unless otherwise indicated, data are the number of lesions with the percentage in parentheses

*BI-RADS* Breast Imaging Reporting and Data System

n^*^ Represents the number of participants

^a^Data are means ± standard deviation

### Differences in ultrafast DCE-MRI parameters between benign and malignant lesions

**Fig. 3:**
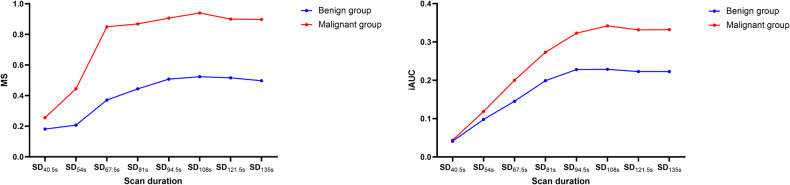
Line plots of ultrafast DCE-MRI parameter values in relation to scan duration for all benign lesions (blue) and malignant lesions (red). MS maximum slope, iAUC initial area under the curve in 60 s

As shown in Table 3 and Fig. 3, ultrafast DCE-MRI parameters varied between benign and malignant lesions across different scan durations. Malignant lesions consistently exhibited higher MS and iAUC values than benign lesions. At SD_40.5s_, the mean MS in malignant lesions was significantly higher than in benign lesions (0.3 ± 0.3 vs 0.2 ± 0.3, *p* = 0.03). No differences were observed in iAUC between benign and malignant lesions (0.04 ± 0.04 vs 0.04 ± 0.04, *p* = 0.75). From SD_54s_ onwards, both the MS and iAUC values of the malignant group were consistently and significantly larger than those of the benign group across all scan durations (all *p* < 0.05).

**Table 3:** Comparison of multiple ultrafast DCE-MRI parameters between benign and malignant breast lesions at eight scan durations

Parameter	Benignancy, (*n* = 55)	Malignancy, (*n* = 96)	*p* value
SD_40.5s_			
MS	0.2 ± 0.3	0.3 ± 0.3	0.03
iAUC	0.04 ± 0.04	0.04 ± 0.04	0.75
SD_54s_			
MS	0.2 ± 0.3	0.4 ± 0.5	< 0.001
iAUC	0.09 ± 0.07	0.1 ± 0.07	0.02
SD_67.5s_			
MS	0.4 ± 0.3	0.8 ± 0.5	< 0.001
iAUC	0.1 ± 0.1	0.2 ± 0.1	0.001
SD_81s_			
MS	0.5 ± 0.3	0.9 ± 0.4	< 0.001
iAUC	0.2 ± 0.1	0.3 ± 0.1	0.001
SD_94.5s_			
MS	0.5 ± 0.3	0.9 ± 0.4	< 0.001
iAUC	0.2 ± 0.1	0.3 ± 0.1	< 0.001
SD_108s_			
MS	0.5 ± 0.3	0.9 ± 0.4	< 0.001
iAUC	0.2 ± 0.1	0.3 ± 0.1	< 0.001
SD_121.5s_			
MS	0.5 ± 0.3	0.9 ± 0.4	< 0.001
iAUC	0.2 ± 0.1	0.3 ± 0.1	< 0.001
SD_135s_			
MS	0.5 ± 0.2	0.9 ± 0.4	< 0.001
iAUC	0.2 ± 0.1	0.3 ± 0.1	< 0.001

Note: unless otherwise indicated, data are presented as means ± standard deviation

*DCE-MRI* dynamic contrast-enhanced MRI, *SD* scan duration, *MS* maximum slope, *iAUC* area under the curve for the initial 60 s

^*^iAUC at SD_40.5s_ and SD_54s_ were referred to as the area under the curve for the initial 40.5 s and 54  s, respectively

*p* values for differences were calculated using the Student’s *t* test or Mann–Whitney *U* test

### Changes in ultrafast DCE-MRI parameters with different scan durations

**Fig. 4:**
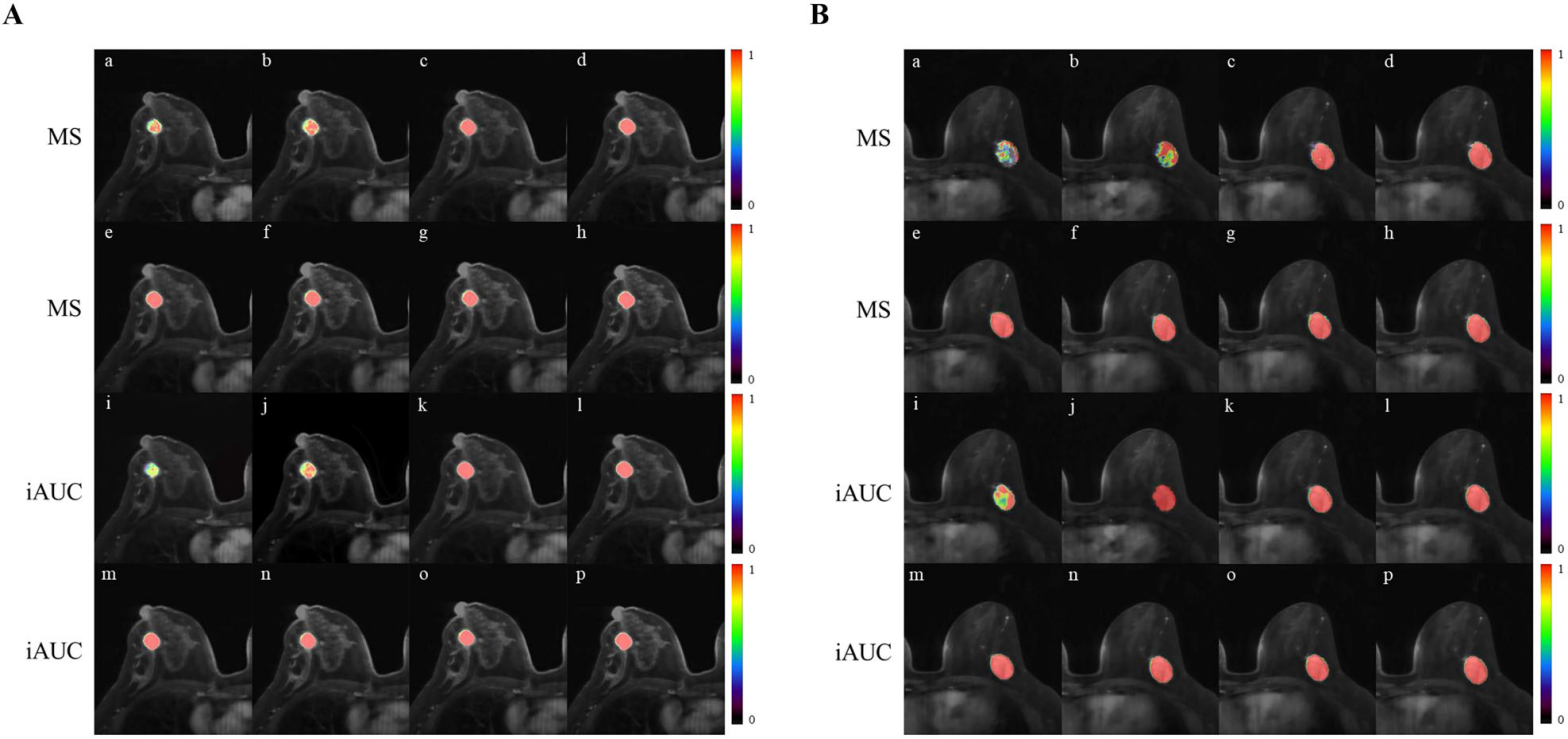
**A** Ultrafast breast DCE-MRI scans in a 49-year-old woman with pathologically confirmed benign fibroadenoma in the right breast. The first two rows (a–h) show color maps of the MS from SD_40.5s_ to SD_135s_. The last two rows (i–p) show color maps of iAUC from SD_40.5s_ to SD_135s_. **B** Ultrafast breast DCE-MRI scans in a 53-year-old woman with pathologically confirmed invasive ductal carcinoma in the left breast. The first two rows (a–h) show color maps of the MS from SD_40.5s_ to SD_135s_. The last two rows (i–p) show color maps of the iAUC in 60 s from SD_40.5s_ to SD_135s_. MS maximum slope, iAUC initial area under the curve in 60 s, SD scan duration

Figures 3 and 4 illustrate the trends in ultrafast DCE-MRI parameter values for benign and malignant lesions over varying scan durations. For both benign and malignant lesions, MS values increased from SD_40.5s_ to SD_81s_. In benign lesions, MS significantly differs between the initial durations (SD_40.5s_ and SD_54s_) and subsequent ones, and between SD_67.5s_ and SD_108s_ (all corrected *p* < 0.05), except between SD_40.5s_ and SD_54s_, and for all pairs from SD_67.5s_ to SD_135s_ (all corrected *p* > 0.05). Malignant lesions also exhibit significant MS differences between SD_40.5s_, SD_54s,_ and all subsequent scan durations (all corrected *p* < 0.05), with no significant differences for all pairs from SD_67.5s_ to SD_135s_ (all corrected *p* > 0.05). Similar patterns are observed in mean iAUC values. In benign lesions, iAUC significantly differs between the initial durations (SD_40.5s_, SD_54s_, and SD_67.5s_) and subsequent ones (all corrected *p* < 0.05), except between SD_67.5s_ and SD_81s_, and for all pairs from SD_81s_ to SD_135s_ (all corrected *p* > 0.05). Malignant lesions show significant iAUC differences between the initial durations (SD_40.5s_, SD_54s_, SD_67.5s_, and SD_81s_) and subsequent ones (all corrected *p* < 0.05), with no significant differences between SD_81s_ and SD_94.5s_, and for all pairs from SD_94.5s_ to SD_135s_ (all corrected *p* > 0.05) (Table 4).

**Table 4:** Paired comparison test results for the ultrafast DCE-MRI parameters in relation to scan duration

Scan duration		*p* values			
		MS		iAUC	
		Benign	Malignant	Benign	Malignant
SD_40.5s_ vs	SD_54s_	> 0.99	0.02	< 0.001	< 0.001
	SD_67.5s_	< 0.001	< 0.001	< 0.001	< 0.001
	SD_81s_	< 0.001	< 0.001	< 0.001	< 0.001
	SD_94.5s_	< 0.001	< 0.001	< 0.001	< 0.001
	SD_108s_	< 0.001	< 0.001	< 0.001	< 0.001
	SD_121.5s_	< 0.001	< 0.001	< 0.001	< 0.001
	SD_135s_	< 0.001	< 0.001	< 0.001	< 0.001
SD_54s_ vs	SD_67.5s_	0.01	< 0.001	0.04	< 0.001
	SD_81s_	< 0.001	< 0.001	< 0.001	< 0.001
	SD_94.5s_	< 0.001	< 0.001	< 0.001	< 0.001
	SD_108s_	< 0.001	< 0.001	< 0.001	< 0.001
	SD_121.5s_	< 0.001	< 0.001	< 0.001	< 0.001
	SD_135s_	< 0.001	< 0.001	< 0.001	< 0.001
SD_67.5s_ vs	SD_81s_	> 0.99	> 0.99	0.19	< 0.001
	SD_94.5s_	0.08	> 0.99	0.001	< 0.001
	SD_108s_	0.04	> 0.99	0.002	< 0.001
	SD_121.5s_	0.07	> 0.99	0.01	< 0.001
	SD_135s_	0.07	> 0.99	0.003	< 0.001
SD_81s_ vs	SD_94.5s_	> 0.99	> 0.99	> 0.99	0.06
	SD_108s_	> 0.99	> 0.99	> 0.99	0.001
	SD_121.5s_	> 0.99	> 0.99	> 0.99	0.004
	SD_135s_	> 0.99	> 0.99	> 0.99	0.01
SD_94.5s_ vs	SD_108s_	> 0.99	> 0.99	> 0.99	> 0.99
	SD_121.5s_	> 0.99	> 0.99	> 0.99	> 0.99
	SD_135s_	> 0.99	> 0.99	> 0.99	> 0.99
SD_108s_ vs	SD_121.5s_	> 0.99	> 0.99	> 0.99	> 0.99
	SD_135s_	> 0.99	> 0.99	> 0.99	> 0.99
SD_121.5s_ vs	SD_135s_	> 0.99	> 0.99	> 0.99	> 0.99

*DCE-MRI* dynamic contrast-enhanced MRI, *SD* scan duration, *MS* maximum slope, *iAUC* initial area under the curve in 60 s

*p* values were calculated using the paired *t* test or Cochran’s *Q* test and were adjusted by Bonferroni correction

### Performance of the ultrafast DCE-MRI parameter among different scan duration

**Fig. 5:**
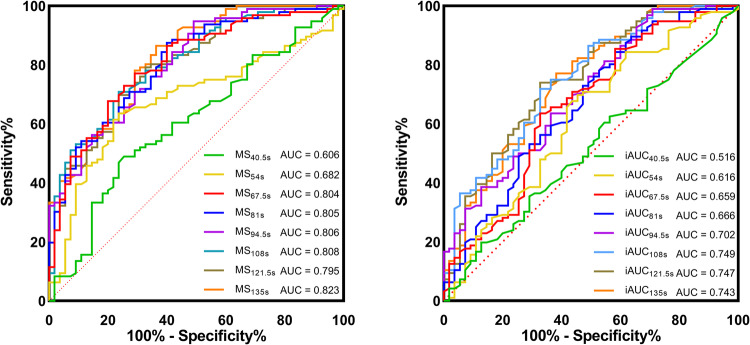
Graphs show the receiver operating characteristic (ROC) curve of ultrafast DCE-MRI parameters with different scan durations. MS_40.5s_ = MS with a scan duration of 40.5 s, MS_54s_ = MS with a scan duration of 54 s, MS_67.5s_ = MS with a scan duration of 67.5 s, MS_81s_ = MS with a scan duration of 81 s, MS_94.5s_ = MS with a scan duration of 94.5 s, MS_108s_ = MS with a scan duration of 108 s, MS_121.5s_ = MS with a scan duration of 121.5 s, MS_135s_ = MS with a scan duration of 135 s. iAUC definitions for different scan durations are the same as MS above. MS maximum slope, iAUC initial area under the curve in 60 s

Table 5 and Fig. 5 depict the AUC values for MS and iAUC across various scan durations. There was a gradual increase in AUC values for both parameters with longer scan durations. The AUC for MS at SD_40.5s_ was significantly lower than its AUC values from SD_67.5s_ to SD_135s_ (all corrected *p* < 0.05). Additionally, MS at SD_54s_ exhibited a significantly lower AUC than its AUC at SD_81s_ (corrected *p* = 0.03). There were no significant differences among AUC pairs for MS from SD_67.5s_ to SD_135s_ (all corrected *p* > 0.05). Similarly, the AUC of iAUC at SD_40.5s_ was significantly lower than that from SD_54s_ to SD_135s_ (all corrected *p* < 0.05). No significant differences in AUC values were observed for iAUC from SD_54s_ to SD_135s_ (all corrected *p* > 0.05) (Tables S1 and S2).

**Table 5:** Diagnostic performance of multiple ultrafast DCE-MRI parameters with eight scan durations

Parameter	Cutoff value	AUC	Sensitivity (%)	Specificity (%)	PPV (%)	NPV (%)	Accuracy (%)
SD_40.5s_							
MS	0.17	0.606 (0.509, 0.698)	48.96 (47/96) [38.95, 59.55]	74.55 (41/55) [62.50, 85.96]	77.05 (47/61) [65.62, 87.04]	45.56 (41/90) [34.44, 55.00]	58.28 (88/151) [50.33, 66.23]
iAUC^a^	0.07	0.516 (0.471, 0.617)	19.79 (19/96) [11.70, 27.47]	87.27 (48/55) [78.12, 96.08]	73.08 (19/26) [55.54, 90.49]	38.40 (48/125) [29.46, 47.20]	44.37 (67/151) [36.42, 52.32]
SD_54s_							
MS	0.18	0.682 (0.590, 0.765)	63.54 (61/96) [53.99, 73.00]	76.36 (42/55) [64.91, 86.79]	82.43 (61/74) [72.88, 90.36]	54.55 (42/77) [43.53, 65.83]	68.21 (103/151) [60.26, 75.50]
iAUC^a^	0.08	0.616 (0.496, 0.711)	69.79 (67/96) [60.00, 79.00]	54.55 (30/55) [43.14, 67.86]	72.83 (67/92) [64.00, 81.32]	50.85 (30/59) [37.87, 64.06]	64.24 (97/151) [56.95, 71.52]
SD_67.5s_							
MS	0.46	0.804 (0.725, 0.875)	77.08 (74/96) [68.37, 84.95]	70.91 (39/55) [58.33, 83.03]	82.22 (74/90) [74.39, 89.77]	63.93 (39/61) [50.85, 75.93]	74.83 (113/151) [67.55, 81.46]
iAUC	0.16	0.659 (0.566, 0.749)	63.54 (61/96) [53.68, 72.73]	67.27 (37/55) [55.00, 79.32]	77.22 (61/79) [68.11, 86.21]	51.39 (37/72) [39.73, 62.69]	64.90 (98/151) [57.62, 72.19]
SD_81s_							
MS	0.46	0.805 (0.737, 0.873)	87.50 (84/96) [80.85, 93.75]	60.00 (33/55) [47.37, 73.59]	79.25 (84/106) [71.57, 87.23]	73.33 (33/45) [60.78, 85.72]	77.48 (117/151) [71.52, 84.11]
iAUC	0.17	0.666 (0.574, 0.752)	84.38 (81/96) [77.42, 91.43]	41.82 (23/55) [29.31, 55.56]	71.68 (81/113) [63.30, 79.47]	60.53 (23/38) [44.73, 76.47]	68.87 (104/151) [61.59, 76.16]
SD_94.5s_							
MS	0.48	0.806 (0.733, 0.870)	94.79 (91/96) [89.90, 98.90]	50.91 (28/55) [38.22, 64.52]	77.12 (91/118) [69.42, 84.03]	84.85 (28/33) [71.42, 96.30]	78.81 (119/151) [72.19, 85.43]
iAUC	0.14	0.702 (0.611, 0.788)	98.96 (95/96) [96.38, 100.00]	29.09 (16/55) [17.77, 42.00]	70.90 (95/134) [62.90, 78.26]	94.12 (16/17) [78.94, 100.00]	73.51 (111/151) [66.23, 80.13]
SD_108s_							
MS	0.70	0.808 (0.733, 0.875)	70.83 (68/96) [61.76, 80.20]	76.36 (42/55) [64.15, 87.50]	83.95 (68/81) [75.94, 91.47]	60.00 (42/70) [48.57, 70.60]	72.85 (110/151) [65.56, 80.13]
iAUC	0.27	0.749 (0.664, 0.828)	71.88 (69/96) [63.11, 80.74]	67.27 (37/55) [54.24, 80.00]	79.31 (69/87) [70.93, 87.34]	57.81 (37/64) [45.71, 69.70]	70.20 (106/151) [63.58, 77.48]
SD_121.5s_							
MS	0.68	0.795 (0.715, 0.867)	79.17 (76/96) [69.87, 87.23]	67.27 (37/55) [54.23, 80.70]	80.85 (76/94) [72.72, 88.75]	64.91 (37/57) [50.98, 77.05]	74.83 (113/151) [67.55, 81.47]
iAUC	0.26	0.747 (0.659, 0.832)	73.96 (71/96) [65.31, 82.56]	67.27 (37/55) [54.39, 79.63]	79.78 (71/89) [70.71, 87.66]	59.68 (37/62) [47.05, 72.42]	71.52 (108/151) [64.22, 79.47]
SD_135s_							
MS	0.56	0.823 (0.750, 0.886)	86.46 (83/96) [79.00, 92.93]	63.64 (35/55) [50.85, 75.86]	80.58 (83/103) [72.47, 87.50]	72.92 (35/48) [59.99, 85.42]	78.15 (118/151) [70.86, 84.11]
iAUC	0.25	0.743 (0.657, 0.827)	77.08 (74/96) [68.48, 85.44]	61.82 (34/55) [48.93, 75.76]	77.89 (74/95) [69.31, 86.08]	60.71 (34/56) [47.37, 74.00]	71.52 (108/151) [64.24, 78.81]

Note: AUC is expressed as a decimal followed by 95% CI in parentheses. Sensitivity, specificity, PPV, NPV, and accuracy are expressed as percentages followed by proportions in parentheses and 95% CI in brackets

*AUC* area under the curve, *CI* confidence interval, *PPV* positive predictive value, *NPV* negative predictive value, *DCE-MRI* dynamic contrast-enhanced MRI, *SD* scan duration, *MS* maximum slope, *iAUC* initial area under the curve in 60 s

^a^iAUC at SD_40.5s_ and SD_54s_ were referred to as the area under the curve for the initial 40.5 s and 54 s, respectively

### Inter- and intra-observer reliabilities for ultrafast DCE-MRI parameters

The ICCs for inter- and intra-observer reliability of the MS and iAUC measurements across eight scan durations were all > 0.70, indicating robust reproducibility of these measurements.

## Discussion

This study was designed to assess the impact of scan duration on parameter estimation and diagnostic performance in breast ultrafast DCE-MRI, aiming to identify the shortest yet effective scan duration for distinguishing between benign and malignant breast lesions. The analysis involved 140 participants who underwent ultrafast DCE-MRI, and we scrutinized the ultrafast protocols acquired over a range of scan durations, from 40.5 s to 135 s. Our findings revealed that at a scan duration of 67.5 s, both MS and iAUC achieved impressive AUCs of 0.804 and 0.659, respectively, for differentiating malignant from benign breast lesions. Notably, starting from SD_67.5s_, both MS and iAUC demonstrated consistent discriminatory capabilities, with no significant difference compared to those obtained at longer scan durations, establishing SD_67.5s_ as the optimal scan duration for this imaging technique.

In this study, significant differences were observed in MS and iAUC between benign and malignant breast lesions starting from a scan duration of 54 s. Malignant lesions exhibited higher MS and iAUC values, indicative of more rapid contrast enhancement compared to benign lesions. MS reflects the rate of contrast agent entry into tumor tissues [23], often associated with higher vascular density and vascular permeability related to neovascularization and inflammatory responses [11]. Similarly, iAUC characterizes the total amount of the contrast agent entering the tumor tissue within the initial 60 s [12] correlating with blood flow, perfusion, and angiogenesis. Elevated MS and iAUC values in malignant breast lesions may signify increased tumor growth and metabolic activity due to heightened demand for blood supply in the tumor tissue. Consistent with our findings, similar conclusions have been reported in previous studies, further supporting the utility of these parameters in distinguishing between benign and malignant breast lesions [11–13].

We observed an increase in both MS and iAUC values with the extension of scan duration, stabilizing around SD_94.5s_. We infer that as the contrast agent perfuses through tissues, the initial enhanced stages of the scan can capture variations in blood flow dynamics and vascular permeability [32]. Notably, stabilizing these parameters after SD_94.5s_ may signify reaching a peak enhancement, indicating that sufficient contrast agents have permeated the tissues. The minor fluctuations in the later stages could be attributed to our study’s manual delineation of VOIs. Manual delineation introduces inherent variability in region selection, contributing to slight deviations in parameter values.

The diagnostic performance of MS in distinguishing benign and malignant breast lesions in our study aligns with previous findings [13, 17, 20]. Honda et al achieved an AUC of 0.76 using CS-VIBE sequences with a 60-s scan duration and a temporal resolution of 3.7 s/phase [20]. Mann et al [13] and Pelissier et al [17] used time-resolved angiography with stochastic trajectories (TWIST)-VIBE sequences with a temporal resolution of 4.32 s/phase and 7.1 sec/phase (scan duration of 78 s and 86.4 s, respectively), respectively, reporting AUCs of 0.94 and 0.829, respectively. In our study, employing CS-VIBE with a 4.5 s/phase temporal resolution, we obtained an AUC of 0.804 with a 67.5-s scan duration. Despite differences regarding parameter settings for ultrafast DCE-MRI protocol, our findings are consistent with prior studies.

Furthermore, our findings suggested that extending scan durations to 81 s and even 135 s did not significantly enhance MS’s diagnostic performance. This underscores the limited benefit of further prolonging scan durations beyond a certain threshold. Utilizing MS derived from the 67.5-s ultrafast DCE-MRI protocol, we identified that about 70.91% of unnecessary biopsies could potentially be avoided. This result demonstrates promise and provides valuable insights for clinical decision-making.

Additionally, it is noteworthy that the majority of malignancies recruited in this study were IDCs, with only a few cases of ductal carcinoma in situ (DCIS) being represented. Therefore, to ensure the homogeneity of the study sample, we focused our analysis solely on IDC. However, it is essential to acknowledge that previous ultrafast DCE-MRI studies in discriminating benign and malignant breast lesions have included a broad spectrum of histological types. Different pathological subtypes of breast cancer exhibit considerable variations in their enhancement characteristics [33, 34]. As such, further work on DCIS and other types of invasive breast cancer breast cancer is required to ensure this timepoint is generalizable to all breast cancer cases.

The ultrafast DCE-MRI parameters are subject to numerous factors, including scan duration, contrast agent characteristics, and technical settings [35–37]. Our study can only offer preliminary insights into optimizing breast ultrafast DCE-MRI protocols, particularly with CS-VIBE techniques and 4.5-s temporal resolution. The result may not be universally applicable. Despite these limitations, the study employed a continuous and frequent acquisition approach with high spatial resolution. This allowed us to determine the impact of scan duration on parameter estimation and diagnostic performance. Meanwhile, the importance of considering the intricacies of scan protocols and the interplay of various technical and biological factors in optimizing ultrafast DCE-MRI for breast lesion characterization must be underscored.

Several limitations in this study must be acknowledged. First and foremost, the study featured a relatively small sample size from a single center, with an uneven sample distribution, and the malignant lesions included only IDCs. A second limitation pertains to the absence of an analysis regarding the influence of different temporal resolutions on diagnostic performance. Understanding how varying temporal resolutions might impact the discrimination of benign and malignant breast lesions could offer valuable insights. Exploring this aspect in subsequent research would be of interest. Thirdly, due to the manual delineation of VOIs, there was an inherent margin of error in the placement and selection of regions for analysis. This can contribute to some degree of parameter value fluctuation. Future studies could focus on reducing this source of variability by implementing more advanced techniques such as automated VOI selection or employing advanced image processing algorithms.

In conclusion, ultrafast DCE-MRI with a scan duration of 67.5 s provides a promising non-invasive method for distinguishing between benign and malignant breast lesions, potentially reducing unnecessary biopsies. However, given the exploratory nature and limited sample size of this study, as well as the inclusion of only IDC as malignant breast lesions, this proposed protocol has yet to be implemented in clinical practice. Future studies involving multi-center, large-sample cohorts that include DCIS and other types of invasive breast cancer are warranted to validate our results.

### Supplementary information

ELECTRONIC SUPPLEMENTARY MATERIAL

## Data Availability

The dataset used or analyzed during the current study is available from the corresponding author upon reasonable request.

## Abbreviations

AUC: Area under the receiver operating characteristic curve
CS: Compressed sensing
DCE-MRI: Dynamic contrast-enhanced magnetic resonance imaging
DCIS: Ductal carcinoma in situ
FOV: Field of view
HER2: Human epidermal growth factor receptor 2
iAUC: Initial area under the curve in 60 s
IDC: Invasive ductal carcinoma
MS: Maximum slope
NPV: Negative predictive value
PPV: Positive predictive value
ROC: Receiver operating characteristic curve
TE: Echo times
TR: Repetition time
VIBE: Volume interpolated breath-hold examination
VOI: Volume of interest
