# Supplementary material for: Optimizing ultrafast dynamic contrast-enhanced MRI scan duration in the differentiation of benign and malignant breast lesions
Source: Insights Imaging. 2024 May 7;15:112. doi: 10.1186/s13244-024-01697-6 (PMC11076431; doi:10.1186/s13244-024-01697-6)
Supplement: Supplementary file 1 — ELECTRONIC SUPPLEMENTARY MATERIAL [file 13244_2024_1697_MOESM1_ESM.pdf]

Optimizing ultrafast dynamic contrast-enhanced MRI scan duration in the differentiation of benign and malignant breast lesions

ELECTRONIC SUPPLEMENTARY MATERIAL

Table S1: *DeLong* test for receiver operator characteristic curve of ultrafast DCE-MRI parameters among different scan duration

Parameter	$MS_{40.5s}$	MS_{54s}	$MS_{67.5s}$	MS_{81s}	$MS_{94.5s}$	MS_{108s}	$MS_{121.5s}$	MS_{135s}
$MS_{40.5s}$	/	> 0.99	**0.002**	**0.002**	**0.003**	**0.003**	**0.01**	**0.002**
MS_{54s}	> 0.99	/	0.10	**0.03**	0.12	0.11	0.63	0.10
$MS_{67.5s}$	**0.002**	0.10	/	> 0.99	> 0.99	> 0.99	> 0.99	> 0.99
MS_{81s}	**0.002**	**0.03**	> 0.99	/	> 0.99	> 0.99	> 0.99	> 0.99
$MS_{94.5s}$	**0.003**	0.12	> 0.99	> 0.99	/	> 0.99	> 0.99	> 0.99
MS_{108s}	**0.003**	0.11	> 0.99	> 0.99	> 0.99	/	> 0.99	> 0.99
$MS_{121.5s}$	**0.01**	0.63	> 0.99	> 0.99	> 0.99	> 0.99	/	> 0.99
MS_{135s}	**0.002**	0.10	> 0.99	> 0.99	> 0.99	> 0.99	> 0.99	/

Note—$MS_{40.5s}$ = MS with a scan duration of 40.5 seconds; MS_{54s} = MS with a scan duration of 54 seconds; $MS_{67.5s}$ = MS with a scan duration of 67.5 seconds; MS_{81s} = MS with a scan duration of 81 seconds; $MS_{94.5s}$ = MS with a scan duration of 94.5 seconds; MS_{108s} = MS with a scan duration of 108 seconds; $MS_{121.5s}$ = MS with a scan duration of 121.5 seconds; MS_{135s} = MS with a scan duration of 135 seconds.
p values were adjusted by *Bonferroni* correction.

Table S2: *DeLong* test for receiver operator characteristic curve of ultrafast DCE-MRI parameters among different scan duration

Parameter	$iAUC_{40.5s}$	$iAUC_{54s}$	$iAUC_{67.5s}$	$iAUC_{81s}$	$iAUC_{94.5s}$	$iAUC_{108s}$	$iAUC_{121.5s}$	$iAUC_{135s}$
$iAUC_{40.5s}$	/	**0.01**	**0.01**	**0.045**	**0.004**	**< 0.001**	**< 0.001**	**0.001**
$iAUC_{54s}$	**0.01**	/	> 0.99	> 0.99	0.93	0.11	0.17	0.18
$iAUC_{67.5s}$	**0.01**	> 0.99	/	> 0.99	> 0.99	> 0.99	> 0.99	> 0.99
$iAUC_{81s}$	**0.045**	> 0.99	> 0.99	/	> 0.99	0.37	0.49	0.63
$iAUC_{94.5s}$	**0.004**	0.93	> 0.99	> 0.99	/	0.77	> 0.99	> 0.99
$iAUC_{108s}$	**< 0.001**	0.11	> 0.99	0.37	0.77	/	> 0.99	> 0.99
$iAUC_{121.5s}$	**< 0.001**	0.17	> 0.99	0.49	> 0.99	> 0.99	/	> 0.99
$iAUC_{135s}$	**0.001**	0.18	> 0.99	0.63	> 0.99	> 0.99	> 0.99	/

Note—iAUC40.5s = iAUC with a scan duration of 40.5 seconds; iAUC54s = iAUC with a scan duration of 54 seconds; iAUC67.5s = iAUC with a scan duration of 67.5 seconds; iAUC81s = iAUC with a scan duration of 81 seconds; iAUC94.5s = iAUC with a scan duration of 94.5 seconds; iAUC108s = iAUC with a scan duration of 108 seconds; iAUC121.5s = iAUC with a scan duration of 121.5 seconds; iAUC135s = iAUC with a scan duration of 135 seconds.

p values were adjusted by Bonferroni correction.
